# Pro/Antioxidant State as a Potential Biomarker of Schizophrenia

**DOI:** 10.3390/jcm10184156

**Published:** 2021-09-15

**Authors:** Dariusz Juchnowicz, Michał Dzikowski, Joanna Rog, Napoleon Waszkiewicz, Kaja Hanna Karakuła, Anna Zalewska, Mateusz Maciejczyk, Hanna Karakula-Juchnowicz

**Affiliations:** 1 Department of Psychiatric Nursing, Medical University of Lublin, 20-124 Lublin, Poland; dariusz.juchnowicz@umlub.pl; 2 1st Department of Psychiatry, Psychotherapy and Early Intervention, Medical University of Lublin, 20-439 Lublin, Poland; michal.dzikowski@umlub.pl (M.D.); kaja.karakula@gmail.com (K.H.K.); hannakarakulajuchnowicz@umlub.pl (H.K.-J.); 3 Department of Psychiatry, Medical University of Bialystok, 16-070 Choroszcz, Poland; napoleon.waszkiewicz@umb.edu.pl; 4 Experimental Dentistry Laboratory and Department of Restorative Dentistry, Medical University of Bialystok, 15-437 Bialystok, Poland; anna.zalewska1@umb.edu.pl; 5 Department of Hygiene, Epidemiology and Ergonomics, Medical University of Bialystok, 15-089 Bialystok, Poland; mateusz.maciejczyk@umb.edu.pl

**Keywords:** biomarkers, schizophrenia, oxidative stress, redox biomarkers, data mining

## Abstract

To allow better diagnosis and management of psychiatric illnesses, the use of easily accessible biomarkers are proposed. Therefore, recognition of some diseases by a set of related pathogenesis biomarkers is a promising approach. The study aims to assess the usefulness of examining oxidative stress (OS) in schizophrenia as a potential biomarker of illness using the commonly used data mining decision tree method. The study group was comprised of 147 participants: 98 patients with schizophrenia (SZ group), and the control group (n = 49; HC). The patients with schizophrenia were divided into two groups: first-episode schizophrenia (n = 49; FS) and chronic schizophrenia (n = 49; CS). The assessment included the following biomarkers in sera of patients: catalase (CAT), glutathione peroxidase (GPx), superoxide dismutase-1 (SOD-1), glutathione reductase (GR), reduced glutathione (GSH), total antioxidant capacity (TAC), ferric reducing ability of plasma (FRAP), advanced glycation end products (AGEs), advanced oxidation protein products (AOPP), dityrosine (DITYR), kynurenine (KYN), N-formylkynurenine (NFK), tryptophan (TRY), total oxidant status (TOS), nitric oxide (NO) and total protein. Maximum accuracy (89.36%) for distinguishing SZ from HC was attained with TOS and GPx (cut-off points: 392.70 and 15.33). For differentiating between FS and CS, the most promising were KYN, AOPP, TAC and NO (100%; cut-off points: 721.20, 0.55, 64.76 and 2.59). To distinguish FS from HC, maximum accuracy was found for GSH and TOS (100%; cut-off points: 859.96 and 0.31), and in order to distinguish CS from HC, the most promising were GSH and TOS (100%; cut-off points: 0.26 and 343.28). Using redox biomarkers would be the most promising approach for discriminating patients with schizophrenia from healthy individuals and, in the future, could be used as an add-on marker to diagnose and/or respond to treatment.

## 1. Introduction

For many years, psychiatric research has been concerned with finding and implementing clinical practice biomarkers specific for diagnostic units [1,2]. The common genetic core of many psychiatric disorders makes searching for factors related strictly to specific diseases appear to be a huge challenge. In recent years, a growing number of articles indicate many potential variables engaged in the pathophysiology of mental disorders, including schizophrenia. Despite this significant expansion of knowledge, the amount of evidence and the strength of the found factors are still insufficient to determine biomarkers, particularly for schizophrenia [3]. Many of the promising results are contradicted by others. According to its definition, a biomarker must be measurable and must indicate a normal, pathological process or response to some intervention [4]. This term includes a broad range of factors, including variables measured from blood, blood–brain barrier, saliva, the neuroimaging testing results or many others [3]. When searching for biomarkers properly, a researcher should take into account non-presenting being a difficulty in assessment. The examination should be quick, cheap and generally available. The determination of solid clinical features which relevantly classify the problematic parts in psychiatry (e.g., the diagnosis) is a big challenge in modern medicine. Huge progress in advanced methods (i.e., neuroimaging, biochemical, molecular, genetic assessment) allows for the determination of even more factors that complicate the picture of the pathological processes of schizophrenia [5].

Biomarkers have been seen as a supplement to diagnosis and cannot replace the current use of the interview-based method. One substance cannot reflect the complicated clinical picture of the disease. The complexity of the biological pathways leads to interactions between many processes, and one change entails another. Some abnormalities occur only due to the functionality formed in the previous step [3,6]. The pathological process should not be under consideration as one change, but as a common network of mutual dependencies. Schizophrenia has been recognized not only as a disease linked with changes in neurotransmitters of the central nervous system [7], but individuals with schizophrenia also show a disruption in many systems. One of the observed disturbances is pro/antioxidant imbalance [8,9]. There is also evidence that antipsychotic treatment affects the oxidation processes, and greater changes in these markers lead to more substantial psychopathology in patients. This indicates that redox state is a potential biomarker of pathophysiology or treatment and therapeutic target in schizophrenia [10]. There has been no consensus on specific disruption in pro/antioxidant pathways for the schizophrenic process. Inconsistent findings could result from differences in the studies’ design (examined population, methods, etc.) [9]. However, as mentioned above, all body reactions and biochemical processes are connected, and levels of one substance affect others and vice versa. More and more studies propose recognizing some diseases by a set of related pathogenesis biomarkers [11,12]. The big challenge is searching and selecting substances tested in research as potential biomarkers [5]. The complexity and heterogeneity of schizophrenia make none of the markers a stand-alone, reliable assay because of either low sensitivity or low specificity. The identification of additional biomarkers is essential for increased relevancy. The decision should not be based on selecting random markers but identifying them by a well-established method. The measurement without establishing a cut-off point is impractical in the clinical approach. Thus, it is necessary to decide on these values for continuous biomarkers [13].

This study aims to assess the usefulness of examining redox biomarkers (RB) disruption in schizophrenia as a potential biomarker of illness.

## 2. Materials and Methods

### 2.1. Study Population

We recruited 147 participants between 18 and 65 years old. The study population consisted of 98 patients who have schizophrenia (SZ group) diagnosed according to DSM 5 criteria [14], which were divided into two equal subgroups: first-episode schizophrenia (first treatment contact) (FS group; n = 49) and chronic schizophrenia (CS group; n = 49).

The exclusion criteria for all groups included the diagnosis of neurological diseases, intellectual disability, organic brain dysfunction, autoimmune diseases or other diseases in unstable phase or addiction (except nicotine and caffeine), and clinical signs of inflammation (high-sensitivity C-reactive protein; hsCRP > 5 mg/mL). Healthy individuals with psychiatric diagnoses were also excluded from entering the study.

The Ethics Committee approved the design of this study of the Medical University of Lublin in Lublin, Poland (project identification code: KE-0254/231/2013). The study complied with the Declaration of Helsinki, and informed consent was obtained from participants.

### 2.2. Data Collection

The assessment was performed one time, and obtained data included socio-demographic and clinical information, as well as blood samples.

#### 2.2.1. Biochemical Procedures

Blood samples (20 mL) were obtained after overnight fasting, and were put into Monovette blood collection tubes (S-Monovette^®^ Serum). Next, samples were centrifuged, and the obtained serum was aliquoted and frozen at −80 °C until analysis.

The concentration of RB was assessed according to earlier protocol, see: [15].

Briefly, we examined the concentration of the following enzymatic and non-enzymatic antioxidants: catalase (CAT), glutathione peroxidase (GPx), superoxide dismutase-1 (SOD-1), glutathione reductase (GR), reduced glutathione (GSH), total antioxidant capacity (TAC), and ferric reducing ability of plasma (FRAP). We also examined the concentration of the following oxidative damage products: advanced glycation end products (AGEs), advanced oxidation protein products (AOPP), dityrosine (DITYR), kynurenine (KYN), N-formylkynurenine (NFK), tryptophan (TRY), and total oxidant status, (TOS). Additionally, we examined concentrations of nitric oxide (NO) and total protein.

The activity of CAT (EC 1.11.1.6), SOD-1 (E.C. 1.15.1.1), GPx (EC 1.11.1.9), GR (EC 1.6.4.2.), GSH, TAC, FRAP, AOPP, TOS, NO were assessed colorimetrically. The content of AGEs, DITYR, KYN, NFK, and TRY were analyzed fluorimetrically. To determine total protein concentration, we used a commercial kit Thermo Scientific PIERCE BCA Protein Assay (Rockford, IL, USA) according to manufacturer instructions. The bicinchoninic acid (BCA) method was applied. All measurements were performed in duplicate samples and standardized to 100 mg of total protein. Reagents were obtained from Sigma-Aldrich, Darmstadt, Germany.

#### 2.2.2. Socio-Demographic and Clinical Data

The collected data included socio-demographic (age, gender) and clinical information (co-occurring diseases, medication taken, duration of illness (in SZ group)). The well-trained physician [M.D] assessed the severity of psychopathological symptoms using the Positive and Negative Symptom Scale (PANSS) in Polish adaptation [16]. The equivalents of antipsychotic medication were calculated based on defined daily doses (DDDs) presented by the World Health Organisation’s Collaborative Center for Drug Statistics Methodology and were presented to 1 mg olanzapine [17]. 

### 2.3. Statistical Analysis

We analyzed the obtained results using Statistica 13 software (TIBCO Software Inc., CA, USA, Palo Alto). The Shapiro–Wilk test indicated the non-Gaussian distribution of most examined factors. As a result, we used non-parametric statistics to characterize participants enrolled in the study. To assess the differences in socio-demographic data between groups, the Man–Whitney U test was applied.

To find a potential panel of biomarkers of SZ for both first-episode and chronic SZ, we applied the classification tree model method (C&RT). C&RT is the most often used method for exploring data. The data-mining analysis allows for determining the roles of examined factors and their hierarchy by categorizing patient subgroups. C&RT divides analyzed data into two subsets in the variable hierarchy order, creating a subset tree. Each of the following subsets is more homogenous than the previous. As we mentioned, the C&RT system is elastic and allows for assessing predictors by considering unequal classification costs, which is impossible using other data algorithms.

Spearman’s rank correlation coefficients were calculated after they were obtained, and suggested biomarkers related to SZ that could determine the potential effect of treatment and psychopathological symptoms on redox state changes.

## 3. Results

### 3.1. Characteristic of Examined Group

Of the 98 individuals in the SZ group, 47 (46.9%) were female, and of the 49 individuals from the HC group, 26 (55.3%) were female (See: Table 1). The median age of the SZ group was 29 and did not differ from the age of the HC group (median: 28). Similarly, the median body mass index (BMI) between the two groups did not differ (median: SZ: 24.5 kg/m^2^, HC 22.8 kg/m^2^). In the patients’ group, the median length of illness was 24 months. The median value of psychopathological symptoms in the SZ group was 87 (points of PANSS) and was different between first-episode patients (median: 104 points) and chronic patients (median: 72 points). The patients’ groups differed in doses of antipsychotic drugs (median olanzapine equivalents in FS was 0, and CS was 17.25). Twenty-six patients from the FS group were drug-naive (53.1%).

### 3.2. Results of C&RT Analysis of Pro/Antioxidant State in Schizophrenia

**Figure 1:**
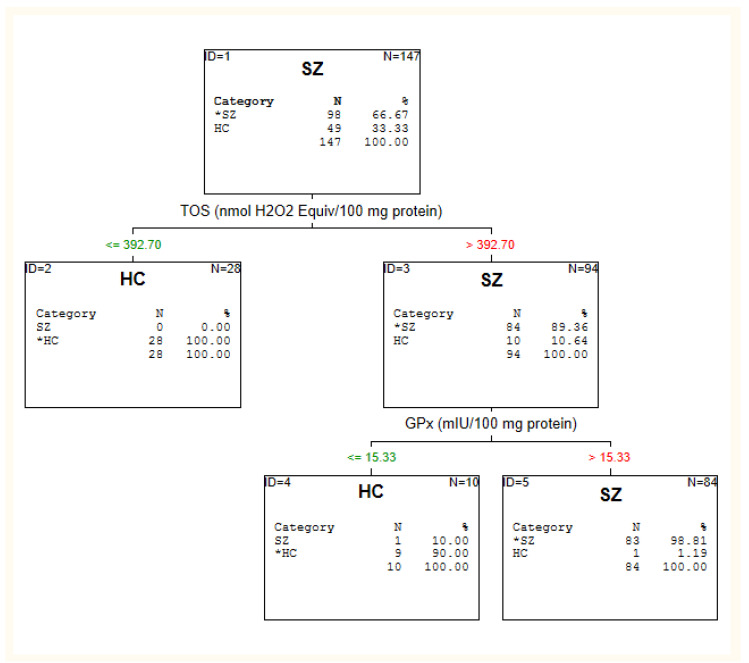
C&RT analysis for healthy individuals (HC) and schizophrenia patients (SZ).

**Figure 2:**
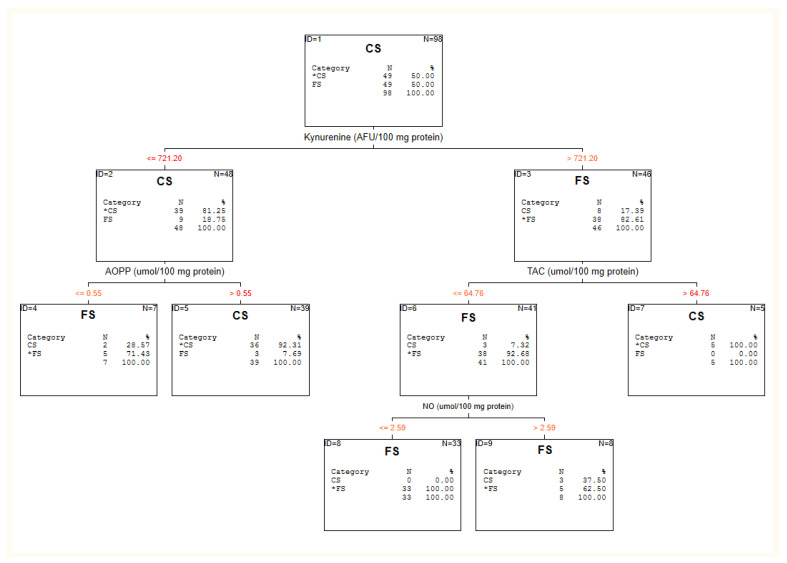
C&RT analysis for first-episode (FS) and chronic patients (CS).

**Figure 3:**
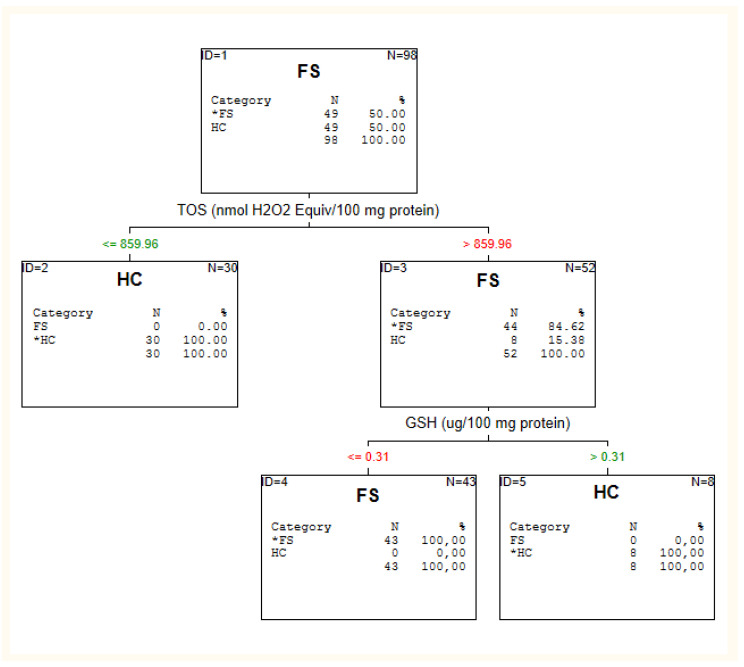
C&RT analysis for healthy individuals (HC) and first-episode patients (FS).

The results of the C&RT analysis are depicted in Figure 1, Figure 2 and Figure 3 and Table 2.

Firstly, we performed an analysis to distinguish the schizophrenia patients sample from healthy individuals, independent of time since diagnosis. 

#### 3.2.1. Schizophrenia Patients vs. Healthy Individuals

Maximum accuracy for schizophrenia diagnosis was attained with two of sixteen candidate biomarkers: TOS and GPx. The cut-off point TOS was >392.70, and GPx was >15.33 for schizophrenia (see: Figure 1). When TOS was taking into account alone, it could classify patients with 89.36% accuracy. Adding GPx to this classification raised the accuracy to 98.81%. Correlation analysis indicates TOS and GPx were not affected by treatment and psychopathological symptoms in SZ patients (*p* > 0.05).

#### 3.2.2. Chronic Schizophrenia vs. First-Episode Patients

The analysis singled out four factors for discriminating first-episode vs. chronic patients: KYN, AOPP, TAC and NO (see: Figure 2). The cut-off point for kynurenine was >721.20 for first-episode patients and helped classify them with 82.61% accuracy from chronic patients. The cut-off point for TAC was 64.76. This split separated the FS patients with 92.68% accuracy. Adding NO (the cut-off point ≤2.59) to the model resulted in the accuracy increasing to 100% for FS patients. Combining lower levels of kynurenine with AOPP below 0.55 strengthened the accuracy of the classification of chronic patients from 81.25% to 92.31%.

Due to some differences in PANSS scale scores and equivalents of antipsychotic medication between CS and FE, we decided to assess the effect of these variables on our results. We found a relationship between olanzapine equivalents and AOPP (R = 0.31; *p* < 0.05); KYN (R = −0.55; *p* < 0.05), TAC (R = 0.36; *p* < 0.05) and NO (R = 0.27; *p* < 0.05). There was also a connection between PANSS total score and KYN (R = 0.38; *p* < 0.05) and TAC (R = −0.31; *p* < 0.05).

#### 3.2.3. First-Episode Schizophrenia Patients vs. Healthy Individuals

Maximum accuracy for distinguishing first episode patients from healthy individuals was attained with two of sixteen candidate biomarkers: TOS and GSH (see: Figure 3). The level of TOS >859.96 had 84.46% accuracy for first-episode patients, and adding GSH to the model (cut-off point below or equal 0.31) allowed for more precise differentiation (100%).

#### 3.2.4. Chronic Schizophrenia Patients vs. Healthy Individuals

**Figure 4:**
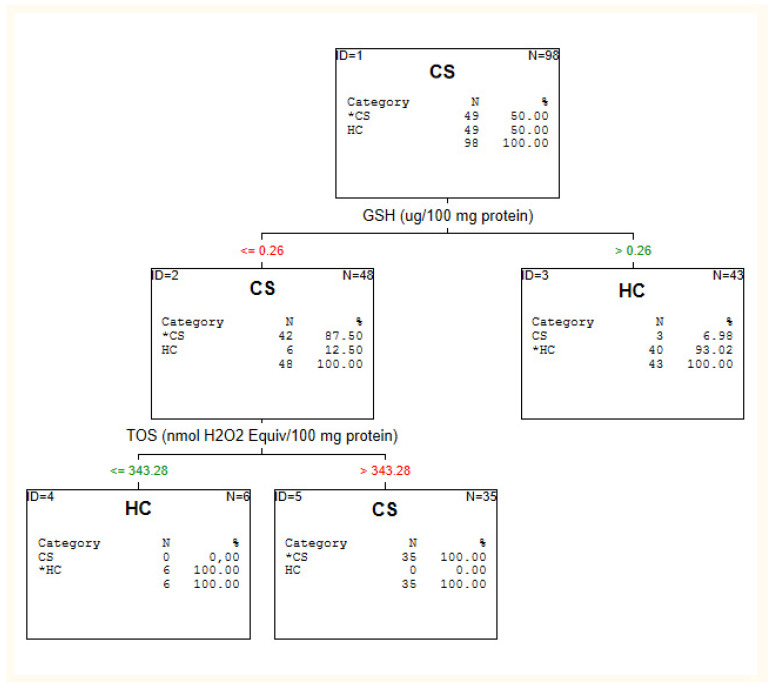
C&RT analysis for healthy individuals (HC) and chronic schizophrenia patients (CS).

For discriminating chronic patients from healthy individuals, GSH and TOS were also the most accurate. The cut-off point for GSH was below or equal to 0.26 with 87.5% accuracy, and adding TOS with the cut-off >343.28 raised the accuracy to 100% (see: Figure 4). The levels of TOS and GSH were not related to treatment and psychopathological symptoms in SZ patients (*p* > 0.05).

## 4. Discussion

The aim of this study was to determine potential biomarkers from the set of redox factors and their cut-off points to distinguish individuals with SZ. Recent research has been focused on multiple possible biomarkers to diagnose different diseases for improving sensitivity and specificity [18,19,20]. In our analysis, we confirmed RB could be considered an additional biomarker of schizophrenia-related biological abnormalities. Despite the high interest in biomarkers, scant attention has been paid to implementing potential biomarkers into clinical practice. Usually, the works concerning diagnostic development are unfamiliar with clinical practice. It should be noted that statistical significance does not reflect clinical relevance [13]. The mean or median in the examined factor does not indicate whether observed differences have clinical utility.

Rapid diagnosis of schizophrenia is still a considerable challenge, and none of the blood factors are enough to be identified as an additional biomarker of SZ. There is an urgent need to find biologically accessible, peripheral biomarkers. Several potential candidates for them have been reported [6]. An increasing number of publications indicate that in searching for biomarkers, automatic machine learning methods are required [21].

In data analysis, the traditional statistical approaches have limited utility in classification [22]. These methods usually do not determine the cut-off points, which distinguish normal and pathological states more precisely. Moreover, classical statistics do not perform the multiple comparisons and calculations that establish interaction or interplay between variables [22]. Making a successful clinical diagnosis in psychiatry is based on a careful patient assessment that includes many factors (e.g., interviews, psychological tests) to eliminate incorrect recognition [23]. It seems impossible to replace detailed examination with one variable from blood or other body fluid tests. All organs and mechanisms of a human organism are connected and dependent on each other. In many clinically essential settings, including the diagnosis of psychiatric disorders, the number of possible etiological variables is quite large [7]. 

A large amount of evidence indicates an important role of pro/antioxidant disturbance in individuals with schizophrenia [9,10,24]. However, the results are inconsistent; researchers may not always examine these same variables, or may do so at different stages of illness. Studies confirm the potential of antipsychotic drugs to decrease RB levels, as well as to decrease the severity of psychopathological symptoms [25,26]. To some extent, this explains the contradictory results of studies and indicates RB as a potential marker of treatment-associated changes and prediction of SZ.

Our analysis showed that the most promising factors in distinguishing schizophrenia patients were TOS and GPx, with 98.81% accuracy. There is a relationship between TOS and negative symptoms, but not all studies supported that TOS is higher in schizophrenia patients [27]. It has been proposed that the neutrophil to lymphocyte ratio (NLR) could reflect RB in patients with schizophrenia. NLR indicates the presence of inflammatory reactions, which are commonly closely related to schizophrenia pathophysiology. Up to now, NLR is more widely used in clinical practice than RB, but its value is correlated positively with TOS [27]. This suggests NLR could be related to negative symptoms and may be helpful in patients with higher NLR levels. However, the issue needs analysis in further investigations.

Kulaksizoglu and Kulaksizoglu have determined the cut-off point for TOS as 5.9 [27]. However, the authors did not include information about units for the given value in their publication. In another study, cut-off for the TOS level was 15.0 μmol H_2_O_2_ Equiv./L [28]. According to Sertan et al., TOS could be considered a marker independent of stage of illness and course of schizophrenia. In the study by Copoglu et al., TOS levels were higher in schizophrenia patients compared to healthy individuals. At the same time, some other (e.g., total antioxidant status and 8-hydroxy-2′-deoxyguanosine) RB markers were more elevated only in the non-remission subgroup. However, TOS levels were significantly higher in remitted patients than in the acute stage of illness [29].

Some studies indicate lower Gpx is related to SZ pathophysiology and a worse course of disease [30]. In our analysis, adding Gpx to TOS further strengthened the effect of schizophrenia classification in the C&RT method. It has been shown that Gpx is affected by treatment, and responders have displayed more “normalized” (higher) enzyme levels. GPx levels decrease during treatment of risperidone, especially in non-responders to therapy [31]. The increased level of Gpx may be a compensatory mechanism during pathophysiological states [31]. On the other hand, two studies have shown that Gpx levels were lower in long-term hospitalized patients treated with risperidone [32,33].

Gpx is crucial in the GSH system, in which changes were observed in chronic and FS patients in our study. The GSH is a non-enzymatic antioxidant that exists at high intracellular levels. The GSH system can counteract the excitotoxicity effect of glutamate and unfavourable oxidative changes in the CNS. The higher brain GSH was accompanied by the faster response to antipsychotics treatment in the drug-naïve first-episode group, and higher levels of GSH reversed the negative impact of hyperglutamatergic state in patients with schizophrenia [34,35]. The relationship between GSH and negative symptoms was confirmed in some studies [36,37]. In our study, the GSH has been helpful to distinguish both patients with first-episode (≤0.31) and chronic patients (≤0.26) from healthy individuals, but the cut-off point was different for these subgroups. More substantial changes were observed in chronic patients, which suggest a gradual decrease in the GSH during illness. According to meta-analysis, patients with schizophrenia have glutathione pathway metabolism dysregulations such as glutathione deficits and abnormalities in the glutathione redox cycle, including lower levels in central and peripheral GSH [38]. 

Blood GSH can cross the blood–brain barrier. Thus, plasma or serum GSH may reflect central GSH. Some authors have also suggested that there might be a relationship between NMDA and GSH. GSH deficits may induce NMDA receptor hypofunction. On the other hand, synaptic NMDA receptor activity boosts and manages GSH metabolism [35,39]. In the mentioned meta-analysis, the changes of the antioxidant level were independent of psychiatric treatment, but the effect of psychiatric treatment on GSH levels are conflicting. Lower levels were more significant in unmedicated patients than in medicated patients. Reduced GSH may contribute to disrupted myelination, which may, in turn, affect clinical symptoms such as cognitive impairment in patients with SZ [38,40]. Some studies indicate correlations between lower GSH levels and greater severity of clinical symptoms, including cognitive dysfunction, while others do not support these results [38,41]. Some results suggest that the age-related decrease in GSH levels in patients with SZ is more significant than in healthy persons, supported by our findings. The role of GSH in the development and severity of pathological schizophrenia symptoms requires explanation [38]. Nevertheless, adding GSH to laboratory tests of patients with psychotic symptoms could confirm an uncertain diagnosis.

In comparing the first episode to chronic schizophrenia, we found four candidates: KYN, AOPP, TAC and NO. We proved lower KYN was related to a chronic state, inconsistent with meta-analysis from 2021 [42]. In the review by Cao et al., lower KYN was found in medication-free persons with SZ, and higher levels were observed after antipsychotic treatment. However, the model studies indicate higher levels of KYN as a factor associated with SZ pathophysiology [43]. In the meta-analysis, the KYN did not differ between healthy individuals and patients with schizophrenia, similar to our results. However, serum levels (blood fraction measured by us, as well) were higher than in the control [42].

In our analysis, PANSS scale scores and equivalents of antipsychotic medication were related to RB biomarkers, especially KYN (R = −0.55; *p* < 0.05 for antipsychotic equivalents and R = 0.38; *p* < 0.05 for PANSS) and TAC (R = 0.36; *p* < 0.05 for antipsychotic equivalents and R = −0.31; *p* < 0.05 for PANSS). Inverse relationships (between PANSS and equivalents for mentioned variables) suggest KYN and TAC could be considered markers related to psychopathology and/or treatment response. However, the results need further investigation.

We found that AOPP could be helpful in more precisely identifying individuals with chronic schizophrenia, with the cut-off point of 0.55. The AOPP reflects protein oxidation and could promote pro-inflammatory factors production (e.g., leptin, interleukin 8). In one study, higher levels correlated with clinical severity scores in SZ [44].

Similar to our results, in a study assessing TAC in chronic and FS patients, this variable did not differentiate them from healthy individuals. The authors also did not show a difference dependent on the duration of the illness, but the study group was relatively small (42 SZ and 42 HC) [45]. Interestingly, TAC status shifted between night and day in SZ patients, which was not observed in a healthy population. Nevertheless, the TAC in SZ was lower in admission and discharge than in HC. The results indicate that patients at admission had significantly higher TAC levels at midday (as same as the control group), but this difference was not seen at discharge. Authors suggest changes could have been the results of antipsychotic treatment, but this point deserves future study [45]. It was found that approximately 2.5 months is needed to increase TAS levels [46]. In our study, the cut-off point which indicates lower levels were seen in FS. Hypothetically, it may be the result of the shorter time of antipsychotic medication treatment.

The studies that assessed NO concentration in SZ are inconsistent [47,48]. The published 2012 meta-analysis showed higher NO levels, but only in patients under treatment and not in healthy persons. More recent studies showed no differences in the compound level between SZ and healthy individuals and indicated that drug-naïve patients had higher levels of this parameter [48]. In our study, NO was helpful to discriminate chronic schizophrenia compared to the first episode, which could also result from a long time of treatment in the chronic group [49]. The NO production is engaged in a cycle related to NMDA receptor activity. The NO affect both as an anti- and prooxidant, and these changes in SZ could have a causal, resulting, or compensating effect [50]. The studies show that six-week antipsychotic treatment with risperidone increases NO and clinical response among patients not receiving antipsychotic drugs. The changes of NO were seen only in patients with response to treatment [49]. During acute exacerbation of schizophrenia symptoms, the NO levels are higher than in healthy individuals and their levels were slightly positively related to the duration of illness. In patients treated with minocycline, the more substantial decrease in plasma levels of NO metabolites was associated with weaker improvement in negative symptoms [51].

It is possible that only some redox balance factors are related to the pathophysiology of psychiatric disorders. In our study, many RB (CAT, GR, SOD-1, FRAP, AGEs, DITYR, NFK, TYR) were not related to schizophrenia. On the other hand, medication (as a tool to reduce disruption homeostasis linked with illness) could affect these factors’ levels in the blood. However, there are many fundamental issues with no answer in terms of biomarkers of schizophrenia, and which need clarification.

## 5. Limitations 

The limitations of our study must be mentioned. Firstly, many changes in the oxidative system are not specific to psychiatric diseases and have been reported to be involved in the pathogenesis of various (also somatic) chronic diseases [52,53]. The study group was relatively small. It makes it difficult to draw definitive conclusions of the potential utility of proposed C&RT schemes. The small study group also limits the power to detect changes in some of the markers proposed by us. The use of antipsychotic treatments and other medication could also be confounding factors that were not investigated. The patients could also suffer from various somatic conditions and could have received treatment for them. However, excluding individuals with an unstable phase of chronic disease minimizes these confounders on the results presented by us. According to some studies, antioxidant enzymes levels were related to the treatment of antipsychotic medication. However, studies establish that an antioxidant defense appears in the early stage of SZ and is independent of treatment. It should be pointed out that the heterogeneity of the symptoms, remission or exacerbation of psychotic symptoms might have affected the results. It should be added, the pro/antioxidant state is strictly related to dietary factors. Many observational studies were able to show an association between intake of various nutritional compounds and plasma markers of OS [54]. Further investigations should raise this issue.

## 6. Conclusions

In conclusion, we find that using RB would be a promising approach to differentiating patients with schizophrenia from healthy controls and, in the future, could be used as an add-on marker to diagnose and/or respond to treatment. Confirmatory studies with a larger sample size in different stages of illness should be performed to establish utility redox balance-related factors as a biomarker of SZ.

## Figures and Tables

**Table 1:** Characteristics of examined population.

Variable	SZ	HC	*p*-Value
Age, years [M]	29	28	NS
Gender (male) [%]	52 (53.06)	21 (42.86)	NS
BMI, kg/m^2^ [M]	24.5	22.9	NS
Duration of illness, months [M]	24	NA	NA
PANSS total [M]	87 FS: 104; CS: 72	NA	<0.001
Dose of olanzapine equivalents (mg) [M]	10 FS: 0; CS: 17.25	NA	<0.001

SZ—schizophrenia; FS—first-episode schizophrenia; CS—chronic schizophrenia; HC—healthy control; BMI—body mass index, PANSS—Positive and Negative Symptoms Scale, M—median.

**Table 2:** Cut-off point for proposed redox biomarkers.

	SZ	FS	CS
**TOS**	>392.70	>859.96	>343.28
**GPx**	>15.33	NA	NA
**GSH**	NA	≤3.12	≤0.26

TOS—total oxidative stress, GPx—glutathione peroxidase; GSH—reduced glutathione; SZ—schizophrenia; FS—first-episode schizophrenia; CS—chronic schizophrenia; NA—not applicable.
